# Enamel Remineralisation with a Novel Sodium Fluoride-Infused Bristle Toothbrush

**DOI:** 10.3390/dj12050142

**Published:** 2024-05-15

**Authors:** Xiaotian Liu, Chun Lok Bryan Lau, Hao Ding, Jukka Pekka Matinlinna, James K. H. Tsoi

**Affiliations:** 1 Department of Orthodontics, Tianjin Stomatological Hospital, School of Medicine, Nankai University, Tianjin 300041, China; 2 Dental Materials Science, Applied and Oral Science and Community Oral Care, Faculty of Dentistry, The University of Hong Kong, Hong Kong, Chinadinghao@connect.hku.hk (H.D.); jpmat@hku.hk (J.P.M.); 3 Tianjin Key Laboratory of Oral and Maxillofacial Function Reconstruction, Tianjin Stomatological Hospital, Tianjin 300041, China; 4 Division of Dentistry, School of Medical Sciences, University of Manchester, Manchester M13 9PL, UK

**Keywords:** demineralisation, remineralisation, toothbrush, bovine, NaF-infused toothbrush

## Abstract

This study aims to investigate whether toothbrushes with fluoride-infused bristles have any (re)mineralisation effects on bovine enamel. Bovine incisors (N = 160) were extracted, and the buccal side of the crown was cut into dimensions of ~5 mm × 5 mm with a low-speed saw. These specimens were randomly allocated into four groups: half (80 teeth) were stored in demineralising solution (DM), and the other half were stored in deionised water (DW) for 96 h. Then, they were brushed with a force of 2.0 ± 0.1 N for five min with a manual toothbrush with either fluoride-infused (TF) or regular (TR) bristles. Microhardness (Vickers), X-ray diffraction (XRD), energy-dispersive X-ray spectroscopy (EDS), and scanning electron microscopy (SEM) were used to investigate the surfaces of the bovine enamel specimens before and after brushing. Two-way ANOVA was used to analyse the hardness data, and the pairwise comparison method was used to analyse the Ca/P ratio, for each group at α = 0.05. The results show that brushing with either of these toothbrushes increased the Vickers microhardness on DM and DW enamel (*p* < 0.001), whereas hydroxyapatite was revealed in all groups by XRD. The DM samples showed a significant increase (*p* < 0.05) in the Ca/P ratios after brushing with TR and TF. Conversely, under DW conditions, these ratios decreased significantly after brushing. In terms of the F atomic%, TF increased significantly. SEM revealed mineral deposition in the DM groups after toothbrushing. To conclude, toothbrushing effectively induces the microhardness of sound and demineralised enamel, while fluoride-infused bristles might be able to retain fluoride on the enamel surface.

## 1. Introduction

Fluoride addition is one of the most used methods for caries control and prevention [1,2,3]. The most common source of fluoride is from toothpaste use, and it is widespread globally. Almost all commercially available toothpaste contains a certain amount of fluoride, with typical concentrations of 1000 parts per million fluoride (ppm F), ranging from low fluoride at 500 ppm F to high fluoride at 1500 ppm F [4,5] using the compounds of, e.g., sodium fluoride (NaF), stannous fluoride (SnF_2_), or Monofluorophosphate (MFP_2_). Evidence has suggested that high-fluoride toothpastes better protect against caries compared to low-fluoride toothpastes [1,6,7]. However, there is a clear association between the former and the risk of developing fluorosis among children [8,9], mainly due to their misingestion [10,11]. Thus, the US Food and Nutrition Board of the Institute of Medicine issued a daily recommendation of fluoride intake for specific age groups in 1997 [12], with the suggestion that the tolerable fluoride amount increases with age/weight. Nevertheless, studies from different countries over the past 20 years have shown that the amount of toothpaste used by children aged 2–7 was much higher than recommended, estimated at a mean of 0.4 g per brushing [13], indicating a likelihood of a higher concentration of fluoride being ingested per brushing.

The prevention of dental caries has always been the priority in dentistry, as it is more time- and cost-effective than its treatment. At the personal level, the combined use of a toothbrush together with toothpaste is always important because (1) a toothbrush can physically remove the biofilm that excretes acid, which can demineralise enamel and dentine [14], and (2) toothpaste contains fluoride that can remineralise the damaged enamel and dentine [15,16]. At the government level, water fluoridation has been in use for more than 70 years, with the mean amount suggested to cause fluorosis approximately 0.06 (±0.01) mg(F)/kg body weight and an upper limit of 1 ppm [17]. The decline in dental caries accompanied by the rise in the prevalence of fluorosis in communities with both fluoridated and non-fluoridated water have raised public concern about the necessity and efficiency of water fluoridation [18]. Concerns have also been raised about the safety of water fluoridation [19,20]; studies have even related fluoridation with various medical conditions, such as child development impairment [21], an increase in hypothyroidism risk [22], neurotoxicity, and cancer [23,24]. However, adverse effects have been seen with ingestion/water fluoridation doses over the recommended limits. Therefore, a reduction in the concentration of fluoride added into the water supply has been implemented in different communities [20]. Studies also support the fact that there are no chemical or biological differences found between artificially and naturally fluoridated water [25,26]. On the other hand, researchers have found that the increased use of topical fluoride such as fluoride varnish [27] and silver diammine fluoride [28] can significantly reduce the prevalence of dental caries, particularly in non-fluoridated water areas [29].

While topical fluoride application remains the safest and most effective method for preventing dental caries, in this study, novel toothbrushes with sodium fluoride (NaF)-infused bristles were tested to see whether they provide a direct benefit to enamel or at least directly prevent fluorosis-causing toothpaste ingestion. With the aim of this study being to investigate the enamel remineralisation efficacy of toothbrushes with NaF-infused bristles, the null hypothesis was that toothbrushes with regular and NaF-infused bristles have similar effects on remineralising sound or damaged enamel.

## 2. Materials and Methods

### 2.1. Specimen Grouping and Study Design

**Figure 1:**
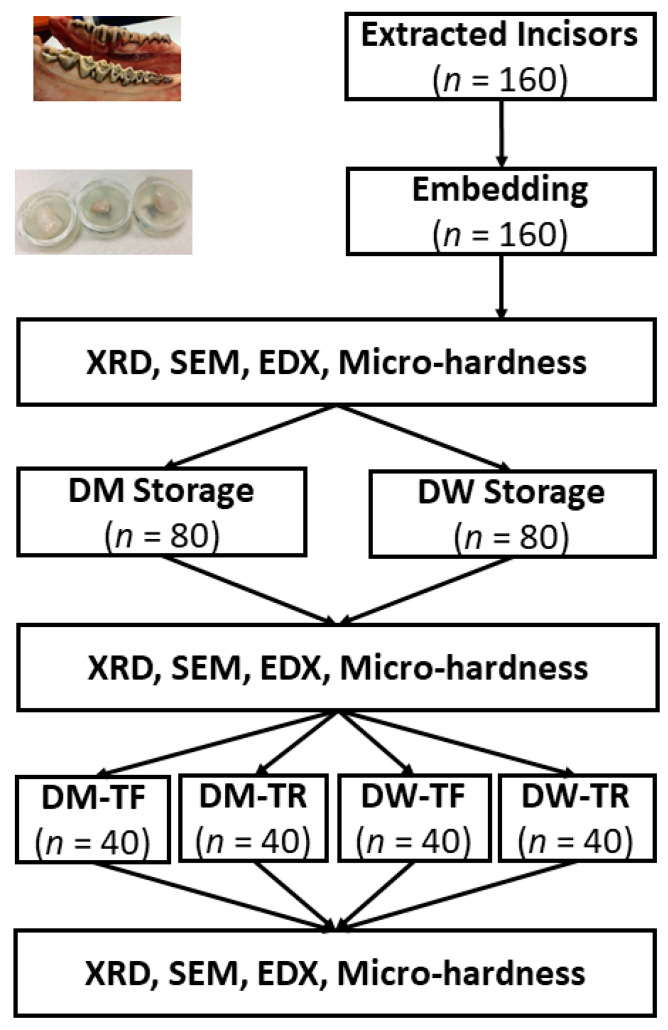
Summary of design, including sample size in each test (*n*). Samples were treated with demineralising solution (DM) or deionised water (DW) and were brushed with either a regular (TR) or NaF-infused toothbrush (TF).

Figure 1 shows a flow chart of this study. Bovine incisors (N = 160) were extracted and the buccal side of the crown was cut into dimensions of ~5 mm × 5 mm with a low-speed saw. Bovine teeth were used because their enamel microstructures are close to those of humans, with a larger flat area [30]. These specimens were randomly allocated into four groups: half (80 teeth) were stored in demineralising solution (DM) and the other half were stored in deionised water (DW) for 96 h. Then, they were brushed for five min with a force of 2.0 ± 0.1 N with a manual toothbrush that had either fluoride-infused (TF) or regular (TR) bristles. Microhardness (Vickers), X-ray diffraction (XRD), energy-dispersive X-ray spectroscopy (EDS), and scanning electron microscopy (SEM) were used to investigate the surfaces of the bovine enamel specimens before and after brushing.

Whole bovine mandible jaws were purchased from a local market after slaughter for human consumption, in agreement with regulations of the Committee on the Use of Live Animals in Teaching and Research (CULATR) of The University of Hong Kong. Incisors were extracted, and soft tissues were debrided from the surface of the enamel and stored in deionised water at 4 °C for less than 3 days before use. No molars/pre-molars were used in this test. The crowns were inspected for hypoplasia, cracks, and white spot lesions, with samples exhibiting these characteristics being excluded; only surfaces that were intact were used.

Poly(methyl methacrylate) (PMMA) rings were used as moulds for embedding specimens, with dimensions of 15 mm in height, 30 mm in diameter, and 2 mm in wall thickness. Polyvinylsiloxane dental laboratory duplicating materials (Elite double 22, Zhermack, Badia Polesine, Italy) and cold-curing denture base material (ProBase^®^, Ivoclar Vivadent Inc., New York, NY, USA) were used for embedding the specimens. The silicone was mixed at a catalyst-to-base ratio of 1:1, and cold-cure PMMA was mixed at a liquid-to-powder ratio of 1:1.5. Both were prepared according to the instructions and followed the recommended setting time suggested by the manufactures.

A reverse technique was used to embed the specimens: The specimen was first placed at the centre of the ring, with the labial surface of the incisor facing downwards; then, a thin layer of silicone slurry was poured in. After the silicone had set, the acrylic mixture was poured in. The thin silicone layer was then removed after the acrylic had set, leaving a window of at least 5 mm × 5 mm of labial enamel exposed on the top, while the lingual side of the enamel was attached to the acrylic. Then, these samples were successively polished with silica carbide (SiC) abrasive paper with numbers 220, 400, 800, and 1200. Each sample was polished for 5 s on papers with different grit sizes. Treatments were then performed after polishing.

### 2.2. Toothbrushes

Toothbrushes with regular and sodium fluoride (NaF)-infused bristles were kindly supplied by DuPont Filaments (Shanghai, China). The bristles were made of Tynex and each toothbrush with NaF-infused bristles contained 2–3 wt% of NaF. The tuft head size was approximately 1.5 cm in length and 8 mm in width. Each specimen was brushed for 5 min while maintaining a force of about ~2.0 ± 0.1 N on the brush head to simulate the force of brushing in an oral environment [31], with a stroke length of 1.5 cm. In addition, about 5 mL of deionised water was applied every 2 min to keep the sample hydrated.

### 2.3. Demineralising Solution

The demineralising buffered solution was prepared with approved chemicals and deionised water, which consisted of 2.2 mM CaCl_2_, 2.2 mM KH_2_PO_4_, and 0.05 M acetic acid; the pH was adjusted to pH 4.4 using 1 M KOH according to Ten Cate and Duijsters [32]. Samples were immersed in the solution (10 mL/sample) for 96 h, which produced artificial carious lesions approximately 100–150 μm in depth. The pH was measured daily using a pH meter and corrected to pH 4.4 with 1 M KOH or 1 M HCl.

### 2.4. Specimen Surface Testing

#### 2.4.1. Microhardness (Vickers)

A total of 80 random samples were analysed for microhardness testing, consisting of 4 groups of 20 samples. A diamond pyramid indenter was used with a Leitz Microhardness Tester (Leitz Inc., New York, NY, USA). The loading time was 10 s with a load of 100 g (0.981 N). The sample size was determined by G*Power software (version 3.1.9.7, Heinrich-Heine-Universität, Düsseldorf, Germany), with Vickers microhardness as the primary outcome, an effect size of 0.4, an α error probability (type I error) of 0.05, and power of 0.8.

#### 2.4.2. X-ray Diffraction (XRD)

One specimen from each group was randomly selected for crystallographic structure analysis. An X-ray diffractometer (Smart Lab, Raigaku, Tokyo, Japan) with continuous scanning was used to obtain data, using medium-resolution Parallel-Beam Cu-Kα radiation (λ = 0.154059 nm) with a tube current and a voltage of 200 mA and 45 kV, respectively. The background spectrum was obtained solely by the specimen holder, and a wafer holder of 3–6 mm was used in all tested samples. Data determination was performed at 15° to 45° two-theta with a 0.05-degree step size at a scanning speed of 3 (equivalent to 20 s per degree). Spectrum analysis was performed using MDI Jade 6 software (Materials Data Inc., Livermore, CA, USA). Crystal peaks were identified manually with the assistance of the joint committee on power diffraction files (JCPDS).

Hydroxyapatite (HAP) and fluorapatite (FAP) standard spectra were obtained from JCPDS (nos. 090432 and 158756). The corresponding crystal lattice peaks were labelled with the corresponding Miller indices (h.k.l).

#### 2.4.3. Energy-Dispersive X-ray Spectroscopy (EDS)

All EDS samples were viewed at a magnification of ×200, the spectrum count was standardised to 20,000 for the elemental spectrum, and the take-off angle of the detector was set at 35°. Samples were observed with the scanning electron microscope (SU1510, Hitachi, Tokyo, Japan) using IXRF Iridium Ultra (IXRF Systems, Inc., Austin, TX, USA) analytical software to analyse the content of carbon (C), oxygen (O), fluorine (F), sodium (Na), phosphorus (P), and calcium (Ca). F atomic % was compared between all groups, and the Ca/P atomic ratio was calculated for each sample based on the identified Ca and P emission lines.

#### 2.4.4. Scanning Electron Microscopy (SEM)

Selected samples before and after toothbrushing were prepared for SEM (SU1510, Hitachi, Tokyo, Japan) imaging by drying in the open air at room temperature for at least 30 min. The dried samples were coated with platinum 80% and palladium 20% in a magnetron sputter (MSP-2S, IXRF Systems, Inc., Austin, TX, USA). Samples were also viewed microscopically with the SU1510 scanning electron microscope (Hitachi, Tokyo, Japan) at various magnifications from ×100 to ×3000.

### 2.5. Statistical Analysis

The mean and standard deviation (mean ± S.D) of the samples from the microhardness (Vickers) tests were calculated. Data normality was assessed by a Shapiro–Wilk test. One-way and two-way analysis of variance (ANOVA) were used to check the main effect of microhardness and interactions between different toothbrushes (TF & TR) and different storing conditions (DW & DM). The Mann–Whitney U and Friedman statistical tests were performed to analyse the statistically significance of the Ca/P ratio and F intensity of different groups from the EDX tests. All statistics were computed using Statistical Package for the Social Science version 23 (SPSS 23) (IBM, Armonk, NY, USA). α = 0.05 was set as the level of statistical significance.

## 3. Results

### 3.1. Microhardness (Vickers)

Table 1 lists the mean microhardness of the samples in all groups. The two-way ANOVA (Table 2) shows that before toothbrushing, all the enamel treated with DW had a higher hardness than the DM samples (*p* < 0.001), and after toothbrushing, in comparison, their microhardness increased significantly (*p* < 0.001), both with TF and TR. However, in both DW- and DM-treated enamel, the difference between TF and TR was not statistically significant (*p* > 0.05).

### 3.2. X-ray Diffraction (XRD)

**Figure 2:**
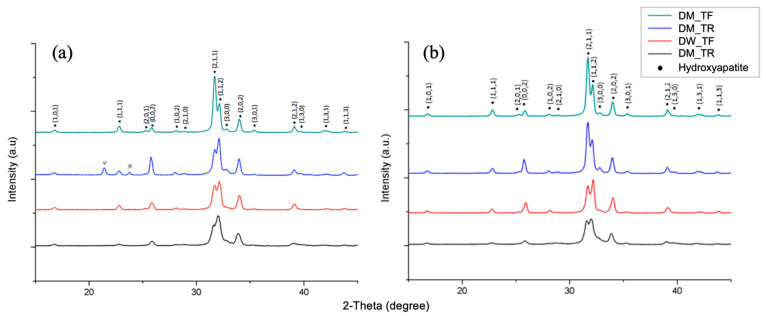
XRD spectrum of samples after treatment with DM and DW (**a**) before and (**b**) after toothbrushing with TF or TR. No changes were found except two impurity peaks (labelled as hollowed inverted triangle and star) in the DM_TR group before toothbrushing.

Crystallographic spectra (Figure 2) were generated for different treatments and conditions, and the results before and after brushing were compared. The HAP standard spectrum obtained from JCPDS confirmed the identity of the generated peaks with matching sharp peaks at 30° to 35° at two-theta peaks of (211), (112), and (202). No obvious difference was found.

### 3.3. Energy-Dispersive X-ray Spectroscopy (EDS)

The Ca and P atomic% were extracted from the analytical software and used to calculate the Ca/P ratio (Table 3); on the other hand, the fluoride atomic% was extracted and compared with the other groups. A non-parametric Friedman test was performed for different conditions, and the DM samples after TR and TF showed a significant increase (*p* < 0.05) in the Ca/P ratios. Conversely, under DW conditions, these ratios decreased significantly after brushing. In terms of the F at% group, TF increased significantly.

### 3.4. Scanning Electron Microscopy (SEM)

**Figure 3:**
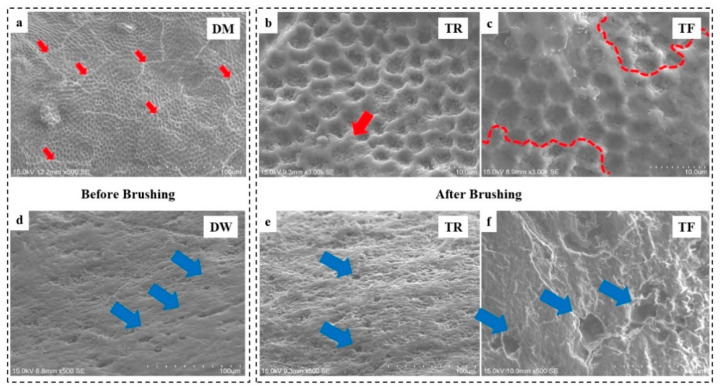
Representative enamel surface images (**a**) after storing in demineralising solution before brushing; after brushing with (**b**) toothbrush with regular bristles and (**c**) toothbrush with fluoride-infused bristles; (**d**) after storing in deionised water before brushing; and after brushing with (**e**) toothbrush with regular bristles and (**f**) toothbrush with fluoride-infused bristles. Red arrows: cracks; blue arrows: pits and holes.

Figure 3a–c show evidence of enamel demineralisation in the DM group, as identified by the ‘fish-scale’-shaped figures, which are the exposed individual enamel rod units. At a magnification of ×3k, the small and lumpy topography within each enamel rod is evidence of tightly packed HAP crystals, whereas cracks are observed on the demineralised enamel surface (Figure 3a, denoted with red arrows). After brushing with TR, a very thin layer of minerals was deposited on the edges of the perikymata (red arrow, Figure 3b). By contrast, a large area of minerals was deposited on the demineralised enamel surface in the DM group using TF brushing (area within the dashed lines, Figure 3c). In the DW group (Figure 3d–f), the morphology is relatively smooth. Only small pits or holes are found on the surface (denoted with blue arrows). No obvious morphological change is observed before and after brushing.

## 4. Discussion

In the current study, the null hypothesis was accepted as remineralization was observed for demineralised enamel after toothbrushing using toothbrushes with either fluoride-infused or regular bristles. However, by using the toothbrush with fluoride-infused bristles, a significantly higher amount of fluoride on the enamel was identified, which has potentials in supplementing fluoride and thus preventing dental caries.

This study revealed that brushing with either a regular or fluoride-infused toothbrush will cause an increase in the hardness of enamel. Tooth enamel was subjected to microhardness testing using a Vickers indenter. A decrease in the hardness of enamel is often associated with carious lesions, while the hardening of enamel is an indication of remineralisation [33,34,35,36]. Bovine enamel was used in this study, but its hardness cannot be directly compared with that of human enamel. Nevertheless, studies have proven that there are no quantitative differences, and these results could act as a model and possibly be extrapolated for use as references in clinical trials [37,38]. The literature recommends the use of Vickers microhardness indentation instead of Knoop indentation in measuring enamel hardness [39]. Because the shape of the indenter is a symmetrical square and can always be conserved, any indentation on a non-flat surface or on dentin can be identified immediately. Meredith and co-workers [40] proposed that Knoop indentation is better, since the indenter produces both long and short diagonals that can cover a wider range of measured material surfaces to give more accurate results. Vickers indentation was used in this study since the indenter was smaller in area compared to Knoop’s, more indentation areas could be taken.

The reported baseline hardness values for bovine enamel range from 299 to 306 VHN [41] and 233 to 326 VHN [42], but the mean hardness values of untreated samples from this study are much higher (467 VHN). Such large variations could be affected by factors such as variations in chemical content [43,44], diet or age, diagonal reading errors, and sample preparation [45]. The above two studies performed a direct comparison with human enamel, without stating whether they were using bovine or human enamel. However, the basic factors, including chemical composition and the remineralisation rate of human (pre-)molars and bovine incisors, are significantly different; thus, these results should only be used for qualitative comparison and should be stated clearly to avoid confusing or misleading readers with the literature. On the other hand, in addition to the hardness test, since toothbrushing may induce surface textural changes, textural and fractal dimension analysis [46,47] may also be useful, particularly to evaluate the changes in mechanical and biological properties after toothbrushing.

The decline in hardness after storing in DM compared to DW was significant (*p* < 0.001). Samples that were brushed with either toothbrush showed an increase in hardness, and the samples brushed with TR had a slightly higher VHN than samples brushed with TF; therefore, the increase in hardness could not be credited to fluoride in this test, despite (1) the EDS showing an uptake of fluoride in the TF groups, and (2) the peak (202) in XRD showing a slightly higher intensity in fluorapatite (FAP) compared to hydroxyapatite (HAP). These results are not significant or unique enough to be identified as FAP instead of HAP. Even so, this proves that the anti-caries agent NaF in the nylon bristles was released. NaF, as an inorganic compound, has high solubility in water (0.987 molal @ 25 °C) [48] and is commonly used in toothpaste, fluoride varnish, and fluoride gel. Fluoride, even at low concentration, can help to thermodynamically form acid-resistant fluorapatite and calcium fluoride. Additionally, an extremely low fluoride level is indeed a catalyst to convert octacalcium phosphate into a tight assembled hydroxyapatite [49]. On the other hand, the uptake of fluoride ion in DW and DM enamel will form a local immobilized store, whereas some fluoride will be released in the presence of acid [50].

The characteristics of HAP peaks were compared with the measured XRD results. Peaks at (211), (112), and (300) are regarded as the peaks that can be most characterised as HAP; however, this study’s results do not agree on these three major peaks, presenting peaks at (211), (112), and (202). The identity of peak (300) was barely identified in all spectra compared to the standard, which had an intensity close to that of peak (112) if not higher (Figure 2). The standard peak obtained from JCPDS includes both HAP and FAP, and there is only slight variation amongst the two. The intensities of the main three peaks remain the same for both apatites.

Toothbrushing caused a decrease in the Ca/P ratio for DW, with an increase in the Ca/P ratio for DM. Indeed, toothbrushing would induce wear on the enamel surface in the DW group, which would obviously damage the intact enamel. Solely using toothbrushing to brush the enamel under water would cause demineralisation, because water would soften the enamel [51], which is why studies would normally remineralise with saliva afterwards. Nonetheless, we did not observe a decrease in hardness, because the EDX measurement used a penetration depth of only 1–2 microns, while the microhardness had an indentation depth of 4–20 microns. This proves that the wear of enamel from the toothbrush is confined to a superficial layer only. For the DM groups, the SEM results (Figure 3a–c) showed that mineral deposition occurs, i.e., remineralisation that is associated with an increase in the Ca/P ratio might occur [52,53].

The limitations of this study are as follows: (1) bovine teeth were used, not human teeth; (2) the NaF infusion percentage was 2–3 wt%, and we should have had different concentrations of NaF so that the release of fluoride could be controlled; and (3) we did not compare the effects of the NaF-infused toothbrush vs. toothpaste. In the future, transmission electron microscopy (TEM) could be incorporated to confirm textures and microstrains in HAP in enamel. X-ray fluorescence (XRF) could also be used to confirm FAP formation. A report has shown FAP regeneration in bone using XRF for element analysis [54]. On the other hand, it might be possible to determine the crystallite size by using the Scherrer equation to understand the microstrains on the apatites, which might affect the hardness of the enamel. In addition, the wear rates from the toothbrush should be assessed to determine the depth of de/re-mineralisation. Nevertheless, a randomised clinical trial after a series of in vitro tests is worth exploring.

## 5. Conclusions

Brushing with either a regular or fluoride-infused toothbrush will cause an increase in the hardness of enamel. In terms of sound enamel, toothbrushing can remove superficial water-softened enamel. Remineralization was observed for demineralised enamel after toothbrushing. Despite the uptake of fluoride by the enamel, FAP was not formed.

## Figures and Tables

**Table 1:** The mean and standard deviation of microhardness of different treatments (before brushing, and after brushing with a regular toothbrush (TR) and with a toothbrush with fluoride-infused bristles (TF)) under different conditions (DW: deionised water, DM: demineralised enamel).

Treatment	Condition	Mean * (VHN)	Std. Deviation
Before Brushing	DW	467.40 ^A^	94.16
	DM	281.04 ^C^	48.88
TF	DW	528.99 ^B^	61.29
	DM	341.40 ^D^	54.75
TR	DW	572.27 ^B^	87.97
	DM	359.63 ^D^	60.39

* Different superscripts indicate statistical significance (*p* < 0.05).

**Table 2:** Two-way ANOVA on the mean microhardness of different groups. Dependent variable: hardness.

Source	Type III Sum of Squares	df	Mean Square	F	Sig.
Corrected Model	1,750,780.258 ^a^	5	350,156.052	68.815	0.000
Intercept	26,024,739.699	1	26,024,739.699	5114.536	0.000
Treatment (before vs. TF vs. TR)	252,108.366	2	126,054.183	24.773	0.000
Condition (DW vs. DM)	1,376,346.367	1	1,376,346.367	270.488	0.000
Treatment × condition	5029.674	2	2514.837	0.494	0.611
Error	783,611.567	154	5088.387		
Total	29,745,514.233	160			
Corrected Total	2,534,391.825	159			
^a.^R Squared = 0.691 (Adjusted R Squared = 0.681)					

**Table 3:** Mean and standard deviation of Ca/P ratio and F at% of different treatments (before brushing, and after brushing with a regular toothbrush (TR) and a toothbrush with fluoride-infused bristles (TF)) under different conditions (DW: deionised water, DM: demineralised enamel).

Treatment	Condition	Ca/P Ratio *	F at% *
Before	DW	1.24 ± 0.10 ^A^	1.26 ± 0.25 ^D^
	DM	1.26 ± 0.12 ^A^	1.39 ± 0.30 ^D^
TF	DW	1.04 ± 0.02 ^B^	2.21 ± 0.52 ^E^
	DM	1.50 ± 0.35 ^C^	2.02 ± 0.44 ^E^
TR	DW	1.04 ± 0.00 ^B^	1.50 ± 0.35 ^D^
	DM	1.44 ± 0.30 ^C^	1.26 ± 0.20 ^D^

* Different superscripts indicate statistical significance (*p* < 0.05) in Friedman’s test.

## Data Availability

The raw data supporting the conclusions of this article will be made available by the authors on request.

## References

[1] Marinho V., Higgins J., Sheiham A., Logan S. (2003). Fluoride toothpastes for preventing dental caries in children and adolescents. Cochrane Database Syst. Rev..

[2] Adair S.M. (2006). Evidence-based use of fluoride in contemporary pediatric dental practice. Pediatr. Dent..

[3] National Institute of Dental and Craniofacial Research (2018). The Story of Fluoridation. https://www.nidcr.nih.gov/health-info/fluoride/the-story-of-fluoridation.

[4] Bijle M.N., Tsoi J., Ekambaram M., Lo E.C.M., Carey C.M., Yiu C.K.Y. (2020). Inter-method reliability for determining total and soluble fluorides in child low-fluoride formula dentifrices. Sci. Rep..

[5] Bijle M.N., Tsoi J., Ekambaram M., Lo E.C.M., Carey C.M., Yiu C.K.Y. (2020). Enhanced Fluoride Bioavailability with Incorporation of Arginine in Child Dentifrices. J. Clin. Pediatr. Dent..

[6] Ammari A., Bloch-Zupan A., Ashley P. (2003). Systematic review of studies comparing the anti-caries efficacy of children’s toothpaste containing 600 ppm of fluoride or less with high fluoride toothpastes of 1000 ppm or above. Caries Res..

[7] O’mullane D., Kavanagh D., Ellwood R., Chesters R., Schafer F., Huntington E., Jones P. (1997). A three-year clinical trial of a combination of trimetaphosphate and sodium fluoride in silica toothpastes. J. Dent. Res..

[8] Narbutaitė J., Vehkalahti M.M., Milčiuvienė S. (2007). Dental fluorosis and dental caries among 12-yr-old children from high-and low-fluoride areas in Lithuania. Eur. J. Oral Sci..

[9] Featherstone J.D. (1999). Prevention and reversal of dental caries: Role of low level fluoride. Community Dent. Oral Epidemiol..

[10] Nakai K.C.A., Rodrigues B.M., Moraes I.F.D., Colombo P.A.R., Juliana J.D.A., Vanessa T., Tercilia G.L., Pelim P.J., Moreira M.M.A.D.A., Rabelo B.M.A. (2011). Factors influencing fluoride ingestion from dentifrice by children. Community Dent. Oral Epidemiol..

[11] Panel M.E. (2007). Topical Fluoride Recommendations for High-Risk Children Development of Decision Support Matrix: Recommendations From Maternal and Child Health Bureau (MCHB) Expert Panel.

[12] Levy S.M., Guha-Chowdhury N. (1999). Total fluoride intake and implications for dietary fluoride supplementation. J. Public Health Dent..

[13] Zohoori F.V., Duckworth R.M., Omid N., O’Hare W.T., Maguire A. (2012). Fluoridated toothpaste: Usage and ingestion of fluoride by 4-to 6-yr-old children in England. Eur. J. Oral Sci..

[14] Ding H., Zhang M., Lo B., Chan K.K.F., Lo E.C.M., Tsoi J.K.H. (2023). A Personalised 3D-Printed Dental Plaque Removal Mouthguard for Older Adults. Int. Dent. J..

[15] Scribante A., Pascadopoli M., Bergomi P., Licari A., Marseglia G.L., Bizzi F.M., Butera A. (2024). Evaluation of two different remineralising toothpastes in children with drug-controlled asthma and allergic rhinitis: A randomised clinical trial. Eur. J. Paediatr. Dent..

[16] Simon L.S., Dash J.K., Philip D.U.S., Sarangi S. (2022). Management of Post Orthodontic White Spot Lesions Using Resin Infiltration and CPP-ACP Materials—A Clinical Study. J. Clin. Pediatr. Dent..

[17] Burt B. (1992). The changing patterns of systemic fluoride intake. J. Dent. Res..

[18] Clark D.C., Hann H.J., Williamson M.F., Berkowitz J. (1993). Aesthetic concerns of children and parents in relation to different classifications of the Tooth Surface Index of Fluorosis. Community Dent. Oral Epidemiol..

[19] Mullen J. (2005). History of water fluoridation. Br. Dent. J..

[20] Kumar J. (2008). Is water fluoridation still necessary?. Adv. Dent. Res..

[21] Lu Y., Sun Z., Wu L., Wang X., Lu W., Liu S. (2000). Effect of high-fluoride water on intelligence in children. Fluoride.

[22] Peckham S., Lowery D., Spencer S. (2015). Are fluoride levels in drinking water associated with hypothyroidism prevalence in England? A large observational study of GP practice data and fluoride levels in drinking water. J. Epidemiol. Community Health.

[23] Chen L., Ning H., Yin Z., Song X., Feng Y., Qin H., Li Y., Wang J., Ge Y., Wang W. (2017). The effects of fluoride on neuronal function occurs via cytoskeleton damage and decreased signal transmission. Chemosphere.

[24] Cantor K.P. (1997). Drinking water and cancer. Cancer Causes Control.

[25] Jackson P., Harvey P., Young W. (2002). Chemistry and Bioavailability Aspects of Fluoride in Drinking Water.

[26] Maguire A., Zohouri F., Mathers J., Steen I., Hindmarch P., Moynihan P. (2005). Bioavailability of fluoride in drinking water: A human experimental study. J. Dent. Res..

[27] Bijle M.N., Ekambaram M., Lo E.C.M., Yiu C.K.Y. (2020). The enamel remineralization potential of fluoride varnishes containing arginine. J. Dent..

[28] Tsoi J., Pun S.Y. (2020). Diamine or diammine. Br. Dent. J..

[29] Network F.A. (1986). The mystery of declining tooth decay. Nature.

[30] Wang C., Fang Y., Zhang L., Su Z., Xu J., Fu B. (2021). Enamel microstructural features of bovine and human incisors: A comparative study. Ann. Anat..

[31] Tan C.M., Tsoi J.K., Seneviratne C.J., Matinlinna J.P. (2014). Evaluation of the Candida albicans removal and mechanical properties of denture acrylics cleaned by a low-cost powered toothbrush. J. Prosthodont. Res..

[32] Cate J.T., Duijsters P. (1982). Alternating demineralization and remineralization of artificial enamel lesions. Caries Res..

[33] Zero D.T., Lussi A. (2005). Erosion—Chemical and biological factors of importance to the dental practitioner. Int. Dent. J..

[34] Devlin H., Bassiouny M., Boston D. (2006). Hardness of enamel exposed to Coca-Cola^®^ and artificial saliva. J. Oral Rehabil..

[35] Maupomé G., Díez-de-Bonilla J., Torres-Villaseñor G., del Carmen Andrade-Delgado L., Castaño V.M. (1998). In vitro quantitative assessment of enamel microhardness after exposure to eroding immersion in a cola drink. Caries Res..

[36] Lussi A., Jaeggi T., Schärer S. (1993). The influence of different factors on in vitro enamel erosion. Caries Res..

[37] Clasen A.B.S., Øgaard B. (1999). Experimental intra-oral caries models in fluoride research. Acta Odontol. Scand..

[38] Mellberg J. (1992). Hard-tissue substrates for evaluation of cariogenic and anti-cariogenic activity in situ. J. Dent. Res..

[39] Gutiérrez-Salazar M.D.P., Reyes-Gasga J. (2003). Microhardness and chemical composition of human tooth. Mater. Res..

[40] Meredith N., Sherriff M., Setchell D., Swanson S. (1996). Measurement of the microhardness and Young’s modulus of human enamel and dentine using an indentation technique. Arch. Oral Biol..

[41] Attin T., Koidl U., Buchalla W., Schaller H., Kielbassa A., Hellwig E. (1997). Correlation of microhardness and wear in differently eroded bovine dental enamel. Arch. Oral Biol..

[42] Tantbirojn D., Huang A., Ericson M., Poolthong S. (2008). Change in surface hardness of enamel by a cola drink and a CPP–ACP paste. J. Dent..

[43] Arends J., Jongebloed W. (1978). Crystallites dimensions of enamel. J. Biol. Buccale.

[44] Schilke R., Lisson J.A., Bauß O., Geurtsen W. (2000). Comparison of the number and diameter of dentinal tubules in human and bovine dentine by scanning electron microscopic investigation. Arch. Oral Biol..

[45] Yassen G.H., Platt J.A., Hara A.T. (2011). Bovine teeth as substitute for human teeth in dental research: A review of literature. J. Oral Sci..

[46] Skoskiewicz-Malinowska K., Mysior M., Rusak A., Kuropka P., Kozakiewicz M., Jurczyszyn K. (2021). Application of Texture and Fractal Dimension Analysis to Evaluate Subgingival Cement Surfaces in Terms of Biocompatibility. Materials.

[47] Paradowska-Stolarz A., Wieckiewicz M., Kozakiewicz M., Jurczyszyn K. (2023). Mechanical Properties, Fractal Dimension, and Texture Analysis of Selected 3D-Printed Resins Used in Dentistry That Underwent the Compression Test. Polymers.

[48] Reynolds J.G., Belsher J.D. (2017). A Review of Sodium Fluoride Solubility in Water. J. Chem. Eng. Data.

[49] Iijima M., Onuma K. (2018). Roles of Fluoride on Octacalcium Phosphate and Apatite Formation on Amorphous Calcium Phosphate Substrate. Cryst. Growth Des..

[50] Cole A.S., Eastoe J.E., Cole A.S., Eastoe J.E. (1988). Chapter 32—Enamel. Biochemistry and Oral Biology.

[51] Zhao D., Tsoi J.K., Wong H.M., Chu C.H., Matinlinna J.P. (2017). Paediatric Over-the-Counter (OTC) Oral Liquids Can Soften and Erode Enamel. Dent. J..

[52] Rodríguez-Vilchis L.E., Contreras-Bulnes R., Olea-Mejìa O.F., Sánchez-Flores I., Centeno-Pedraza C. (2011). Morphological and structural changes on human dental enamel after Er: YAG laser irradiation: AFM, SEM, and EDS evaluation. Photomed. Laser Surg..

[53] Kourkoumelis N., Balatsoukas I., Tzaphlidou M. (2012). Ca/P concentration ratio at different sites of normal and osteoporotic rabbit bones evaluated by Auger and energy dispersive X-ray spectroscopy. J. Biol. Phys..

[54] Nigri E.M., Bhatnagar A., Rocha S.D.F. (2017). Thermal regeneration process of bone char used in the fluoride removal from aqueous solution. J. Clean. Prod..

